# Case Report: Heparin-induced thrombocytopenia during COVID-19 outbreak: the importance of scoring system in differentiating with sepsis-induced coagulopathy

**DOI:** 10.12688/f1000research.52425.1

**Published:** 2021-06-14

**Authors:** Louisa Fadjri Kusuma Wardhani, Ivana Purnama Dewi, Denny Suwanto, Meity Ardiana

**Affiliations:** 1 Faculty of Medicine, Airlangga University, Surabaya, Indonesia; 2 Department of Cardiology and Vascular Medicine, Dr. Soetomo Hospital, Surabaya, Indonesia; 3 Faculty of Medicine, Duta Wacana Christian University, Yogyakarta, Indonesia; 4 Department of Cardiology and Vascular Medicine, Bhayangkara H.S Samsoeri Mertojoso Hospital, Surabaya, Indonesia

**Keywords:** Heparin Induced Thrombocytopenia, HIT, COVID-19, mortality

## Abstract

**Background: **COVID-19 disease is accompanied by derangement of coagulation with a risk of fatal thromboembolic formation. COVID-19 patients are among those indicative for heparin treatment. Increased heparin administration among COVID-19 patients increased heparin induced-thrombocytopenia's risk with/without thrombocytopenia.

**Case presentation: **We present a 71-year-old male patient who came to the emergency department (ED) with a COVID-19 clinical manifestation that PCR nasopharyngeal swab confirmed. He was assessed to have acute respiratory distress syndrome (ARDS), as shown by rapid progression of hypoxemic respiratory failure and bilateral pulmonary infiltrate. He was then treated with moxifloxacin, remdesivir, dexamethasone, heparin pump, and multivitamins. During admission, his respiratory symptoms got worse, so he transferred to the ICU for NIV support. On the ninth day of admission, he had gross hematuria followed by a rapid fall of platelet count. We used two different scoring systems (4Ts and HEP scoring system) to confirm the diagnosis of heparin-induced thrombocytopenia (HIT). Following the discontinuation of heparin injection, the thrombocyte continued to rise, and hematuria disappeared.

**Conclusion: **Heparin-induced thrombocytopenia is associated with an increased risk of severe disease and mortality among COVID-19 patients. The differential diagnosis of HIT could be difficult among COVID-19 patients as thrombocytopenia can also be caused by infection progression. We use two scoring systems, 4Ts and HEP scoring, that can help us to manage the patient. With good management, we can avoid patient morbidity and mortality.

## Background

The outbreak of the novel-coronavirus named SARS-CoV-2 was first identified in Wuhan, China, in January 2020. This was later classified as a pandemic because it affected more than 114 countries with more than two million cases. The infection can be manifested as fever, dry cough, myalgia, diarrhea, and anosmia. The more severe cases can present as acute respiratory distress syndrome (ARDS), as shown by rapid progression of hypoxemic respiratory failure and bilateral pulmonary infiltrate. It can also be manifested as derangement of coagulation that ranges from hypercoagulability to venous thromboembolism (VTE). It has been previously described that 64 out of 150 COVID-19 patients complicated by ARDS developed various thromboembolic complications from ischemic strokes to pulmonary embolism. ^1^ COVID-19 patients have elevated D-dimer concentration, increased fibrin degradation products (FDP), and lower antithrombin levels. These are the reasons for anticoagulation administration among COVID-19 patients. Heparin is the agent of choice for anticoagulation, especially in those with severe COVID-19 manifestation. ^2– 5^

Although heparin is commonly used in COVID-19 patients, its side effects cannot be forgotten. The most avoided side effect, which is potentially fatal with 20% mortality rates, is heparin induced thrombocytopenia (HIT) with/without thrombocytosis. This is caused by the formation of antibodies (IgG) against the complex of platelet factor 4 (PF4) and heparin. The PF4/IgG antibodies complex can activate platelets, which can cause catastrophic thrombosis. It can occur within 5–10 days of heparin therapy in 0.5–3% of patients. It can also develop rapidly within 24 hours after re-exposure of heparin in some patients with a recent heparin administration history. ^2, 6, 7^ An increasing incidence of HIT occurs among COVID-19 patients, explained by exacerbated immune reactions and probably by an increased release of PF4, linked to platelet activation. However, the underlying pathophysiology of increased HIT risk and thrombosis risk in COVID-19 patients is not yet well understood. ^8^ We present a HIT case with severe thrombocytopenia followed by gross hematuria manifestation in a COVID-19 patient.

## Case presentation

A 71-year-old male patient presented to the emergency department (ED) with a chief complaint of dry cough for one week followed by dyspnea that had increased three days before admission. He had a fever one week prior that decreased with NSAIDs, and no other complaints were claimed. He had no contact history with confirmed COVID-19 patients. His past medical illnesses consisted of diabetes mellitus, hypertension, and old myocardial infarction (OMI). He previously consumed aspirin, bisoprolol, lisinopril, diltiazem, and atorvastatin. He also had a history of routine subcutaneous insulin usage.

His blood pressure (BP) was 132/75 mmHg, heart rate (HR) 79 beats per minutes (bpm) regular, and apparent dyspnea with a respiratory rate (RR) of 28 breaths per minute. He had desaturation, with oxygen saturation of 85% (free air) that improved with a non-rebreathing oxygen mask (NRM) 15 Liters per minute to 95%. He was also pyretic (38 ^o^C). His physical examination showed no increased jugular venous pressure, bilateral lung crackles, pleural friction rub, and no leg edema.

Comprehensive evaluations were performed, including ECG, COVID-19 antigen swab, laboratory examination, and chest radiography. His initial ECG showed normal sinus rhythm of 79 bpm, left axis deviation, with inferior OMI. Laboratory data showed increased CRP (195.3 mg/L), hypokalemia (3.2 mEq/L), respiratory failure, and respiratory acidosis. Chest radiography showed cardiomegaly and bilateral pneumonia. COVID-19 antigen swab was positive. Thus patient and family were consent for isolation and tracing procedure. As respiratory failure presented with severe ARDS ( *P*aO _2_/ *F*iO _2_ 82 mmHg) and hypoxemia, the patient was informed about the need for performing an intubation procedure. Since our intensive care unit was occupied, we planned a referral to another facility. The patient and family refused it, so we hospitalized the patient in our lower care unit.

He was then started on antibiotic treatment with moxifloxacin, antiviral therapy with remdesivir, dexamethasone, heparin pump, and multivitamins. He also continued his daily medication (insulin, aspirin, lisinopril, beta-blockers, nitrates, and atorvastatin). Tocilizumab 400 mg was also given on the second day before admission. His vital signs and laboratory examinations were routinely monitored.

During the sixth day of admission, his oxygen saturation deteriorated. He was then transferred to the unoccupied intensive care unit to receive non-invasive ventilation (NIV). The vital signs showed BP 168/87 mmHg, HR 65 bpm regular, and RR 30 breaths per minute. During the following days, the patient's symptoms showed slight improvement. His laboratory examination on the tenth day showed leukocytosis, thrombocytopenia, increased neutrophil–lymphocyte ratio (NLR), increased D-Dimer, and PCR remained positive for COVID-19. No prolonged hemostatic values were found. Plasma prothrombin time (PPT) was 20 sec, and activated partial thromboplastin time (APTT) was 38.6 sec.

**Figure 1.**
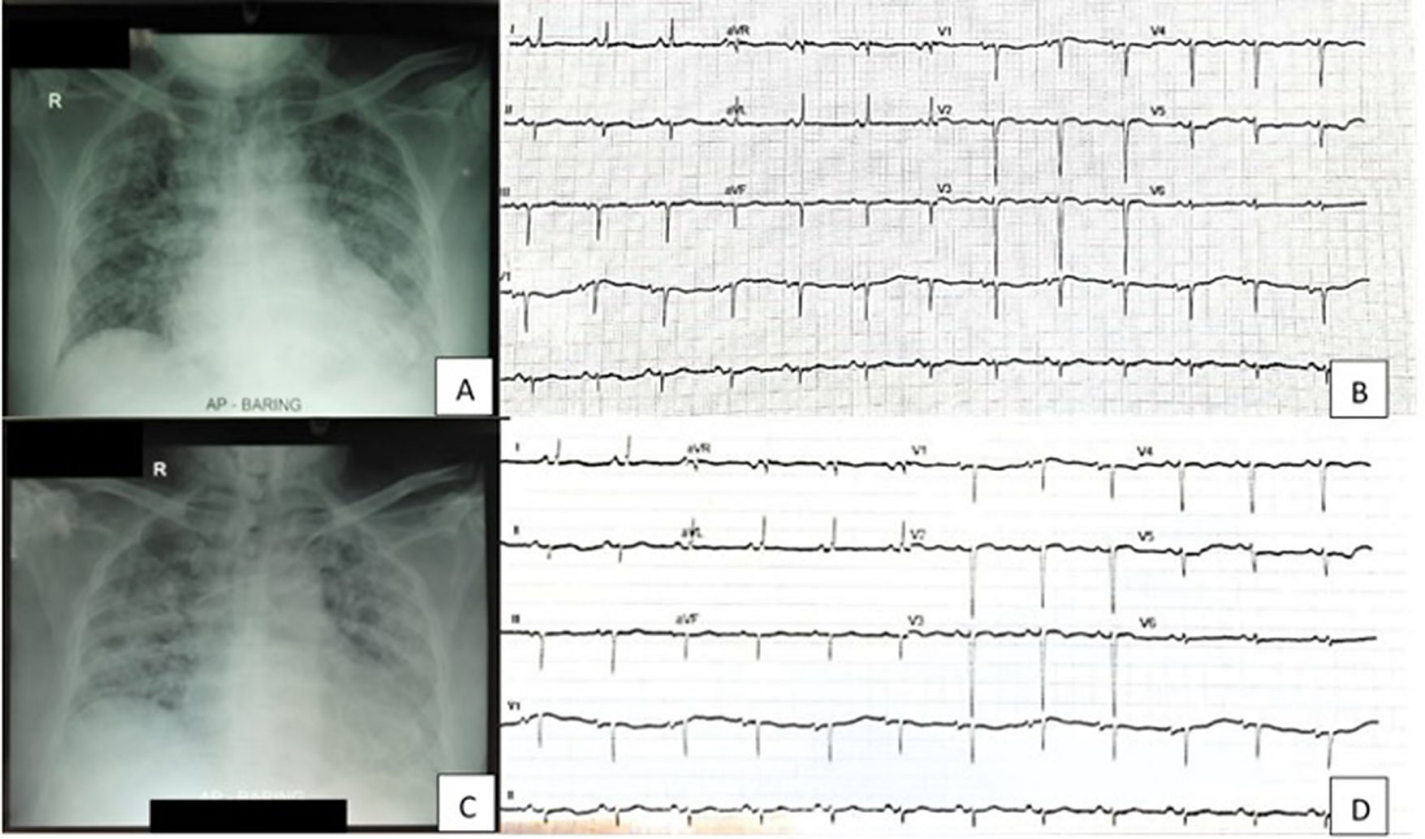
Radiology and electrocardiography (ECG) comparison of patient on first day admission (A, B) and ninth day admission (C, D).

On the ninth day of admission, the patient seemed anxious with complaints of urinary pain. It showed that he had gross hematuria while he was on heparin therapy within targeted APTT. His vital signs showed BP 120/77 mmHg, tachycardia 110 bpm, and tachypnea 30 breaths per minute. Laboratory data showed significantly reduced thrombocytes (40,000 mg/L). Urine examination showed gross hematuria with leukocyturia (10–15 cells/field), nitrituria, and proteinuria (2+). The patient underwent ECG and chest radiography evaluation, which showed no significant changes ( Figure 1).

Heparin was thought to be the underlying cause of thrombocytopenia in this patient. Since heparin antibody was not routinely checked in the developed country, we choose to switch our anticoagulant with rivaroxaban 20 mg twice daily. The symptoms were then improved with the absence of hematuria and reduced dyspnea. The thrombocyte evaluation showed normalization to 177,000 on the eleventh day. The patient was transferred back to the low care unit on the twentieth day of admission. PCR was showed negative one day later, and the patient was then discharged. One month after discharge, the patient returned to the outpatient clinic without symptoms. The patient and family have then suggested the need for routine control for a better outcome after surviving COVID-19 infection.

## Discussion

We report a patient presented to the ED with clinical manifestation of COVID-19 disease that was diagnosed by positive antigen swab for SARS-CoV-2 and confirmed by PCR nasopharyngeal swab the day after. He was also presented with desaturation and severe hypoxemia, leading to severe ARDS ( *P*aO _2_/ *F*iO _2_ of 82 mmHg). COVID-19 patient is among those indicative for heparin treatment. Low-molecular-weight heparin is used as prophylaxis for the thromboembolic complication of COVID-19, while unfractionated heparin is commonly used in severe manifestations.

A routine evaluation of both physical and laboratory examination is needed to evaluate treatment and side effects of infection and therapy of COVID-19. A fatal side effect that can occur during heparin treatment is HIT. HIT is characterized by a fall of platelet count with/without a sign of arterial and venous thrombosis during heparin administration and disappears equally quickly once the heparin is withdrawn. The widely known diagnostic criteria for HIT includes (1) HIT antibodies to PF4-heparinoid complexes, (2) HIT antibodies-mediated platelet activation, (3) progressive platelet fall 40-50% from baseline, (4) thrombocytopenia occurs within 5–10 days after initiation or 24–48 hours after re-exposure of heparin, and (5) thrombosis occurs in patients treated with heparin. It should also be suspected of an unexplained fall in platelet count by 50%, skin lesion at the heparin injection site, and systemic reaction to heparin injection. Thrombocytopenia in HIT is usually moderate to severe, with the fall of platelet rarely <100,000 platelets/μL. It has been reported that 30–60% of patients can suffer venous events while 15–20% can suffer arterial events while bleeding manifestation is rare. ^2, 5, 9, 10^

The differential diagnosis of HIT could be difficult among COVID-19 patients as thrombocytopenia can also be caused by infection progression. Disseminated intravascular coagulopathy (DIC) can complicate the septic status of COVID-19, called sepsis-induced coagulopathy. Thrombocytopenia is associated with an increased risk of severe disease and mortality among COVID-19 patients. On the other hand, heparin exposure is correlated with severe thrombocytopenia, suggesting that HIT may be responsible for severe thrombocytopenia in COVID-19. PF4/H antibodies or a scoring system makes the presumptive diagnosis of HIT. The measurement of PF4/H is not commonly checked in our center, so we use the scoring system to measure the possibility of HIT. ^2, 5, 7, 9, 11^

Our patient experienced a rapid fall of thrombocytes on a ninth day following gross hematuria manifestation. The clinical suspicion laid on HIT as it is known to be a fatal side effect of heparin administration. We use two scoring systems, which showed a high probability of HIT in our patient using the 4Ts (5 points) and HEP scoring (5 points) system ( Figure 2). The use of two different methods reduces the possibility of overdiagnosis since improper anticoagulation in COVID-19 patients can lead to worse prognosis. The 4Ts scoring system valued thrombocytopenia, timing of platelet fall, thrombosis (or other sequelae of HIT), and the absence of other thrombocytopenia cause (medication, DIC) in which the fourth indicator is subjective to the discretion of the observer. On the other hand, the HEP scoring system listed infection as a marker of thrombocytopenia which is found in COVID-19 patients. The clinician can confidently reduce the use of alternative anticoagulation due to false diagnosis in clinically oriented HIT assessment using the HEP scoring system.

**Figure 2.**
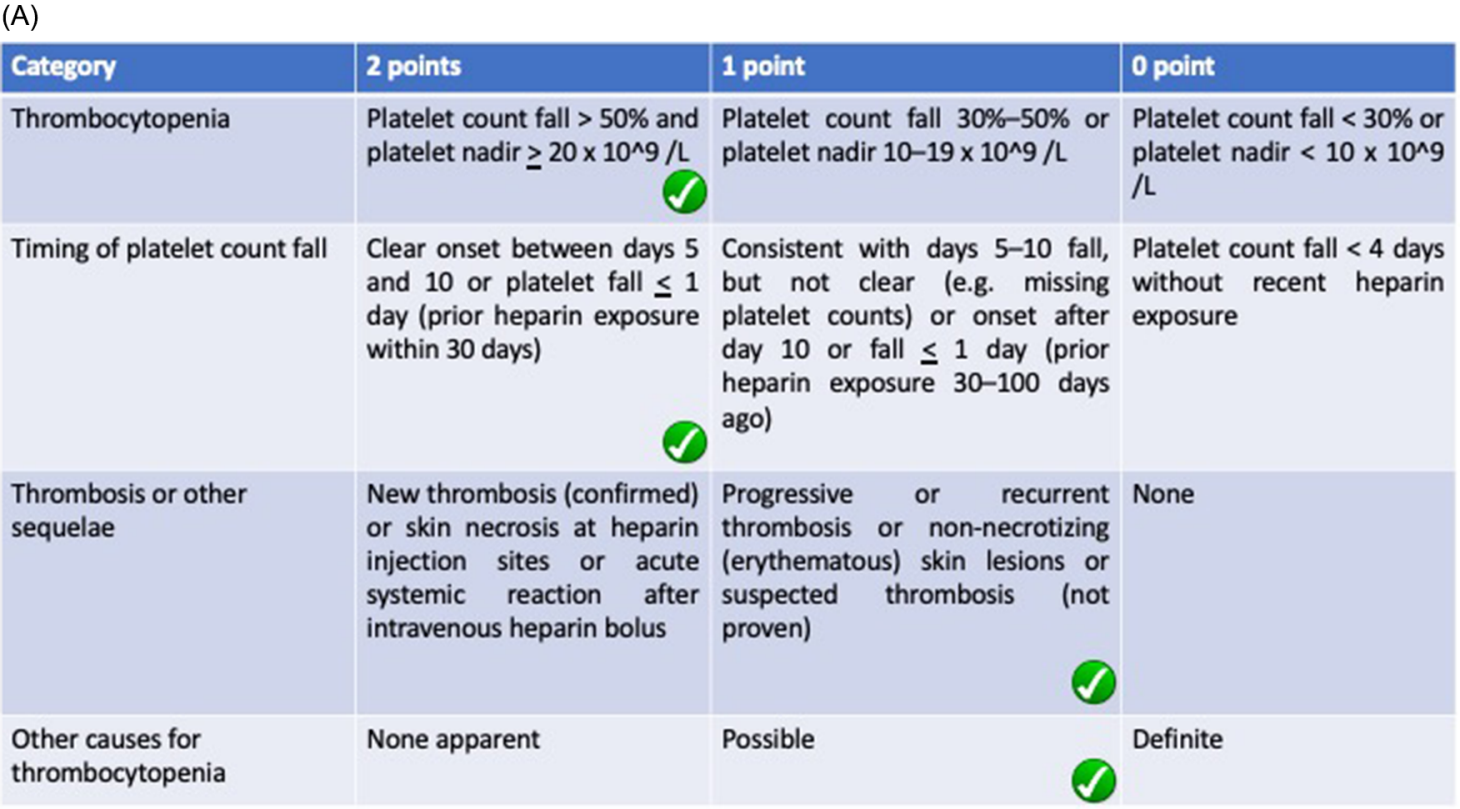
Checklist of scoring system used in measuring HIT probability using (A) 4Ts scoring system showing 6 point and (B) HEP scoring system showing 5 point. Both measurements show high probability of HIT in this patient.

**Figure 2 (continued).**
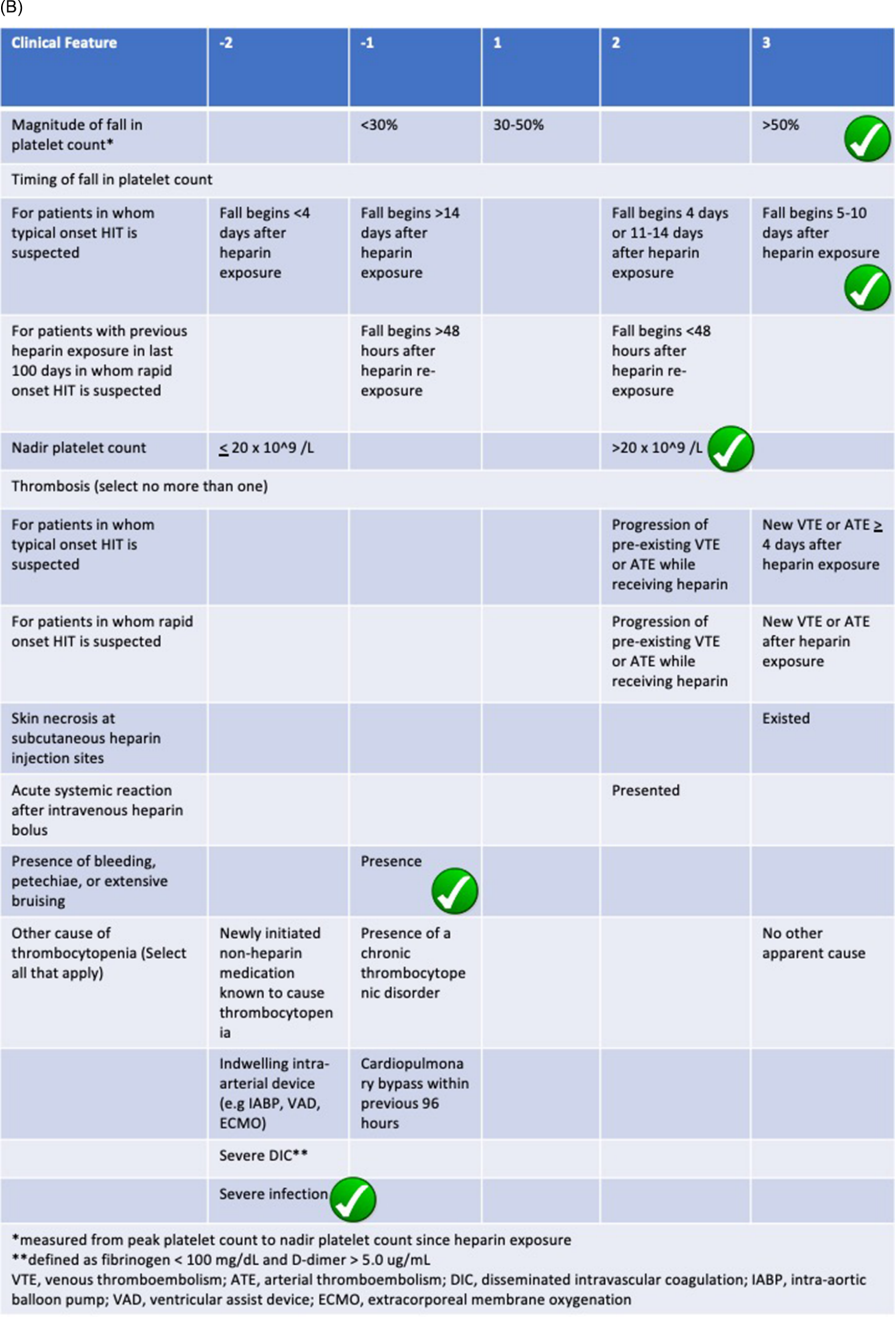


**Figure 3.**
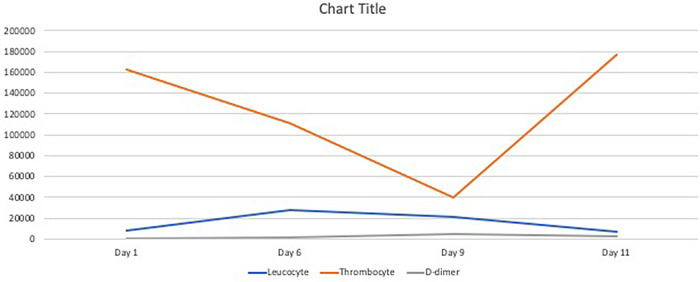
Summary of laboratory results.

When isolated HIT is suspected clinically, the discontinuation of heparin-based therapy is recommended. Clinicians shall consider the initiation of other anticoagulant agents using direct thrombin or factor Xa inhibitors to prevent thrombus formation. ^7, 9^ Following the discontinuation of heparin injection and switching to rivaroxaban, the thrombocyte continued to rise, and hematuria disappeared. It was consistent with HIT in which is rapidly disappeared once heparin administration stopped ( Figure 3).

## Conclusion

There is an increased use of heparin therapy during the outbreak of SARS-CoV-2 infection as it is a known risk of fatal thromboembolic formation. HIT is a rare but fatal complication following heparin administration. As the progression of COVID-19 also shows thrombocytopenia, it is somehow ambiguous to perform discontinuation of heparin. Moreover, the measurement of PF4/H antibodies is not available in most of the centers. It makes the clinical assessment of HIT critical to risk-stratify the need to stop heparin as it also led to poor prognosis. The use of 4Ts scoring system is subjective to the observer's discretion to determine the involvement of other thrombocytopenia. However, the HEP scoring system has multiple values that help physicians determine the HIT risk probability. We used two different scoring systems (4Ts and HEP scoring systems) that show a high HIT probability, so switching therapy to rivaroxaban is initiated.

## Declaration

This case report does not require ethical approval as it is not human or animal research.

## Consent

Written informed consent for publication of their clinical details and/or clinical images was obtained from the patient.

## Data Availability

All data underlying the results are presented within the manuscript, and no additional source data are required.
