# Case Report: Heparin-induced thrombocytopenia during COVID-19 outbreak: the importance of scoring system in differentiating with sepsis-induced coagulopathy

**DOI:** 10.12688/f1000research.52425.2

**Published:** 2021-08-26

**Authors:** Louisa Fadjri Kusuma Wardhani, Ivana Purnama Dewi, Denny Suwanto, Ade Meidian Ambari, Meity Ardiana

**Affiliations:** 1 Faculty of Medicine, Airlangga University, Surabaya, Indonesia; 2 Department of Cardiology and Vascular Medicine, Dr. Soetomo Hospital, Surabaya, Indonesia; 3 Faculty of Medicine, Duta Wacana Christian University, Yogyakarta, Indonesia; 4 University of Indonesia, Jakarta, Indonesia; 5 Department of Cardiology and Vascular Medicine, Bhayangkara H.S Samsoeri Mertojoso Hospital, Surabaya, Indonesia

**Keywords:** Heparin Induced Thrombocytopenia, HIT, COVID-19, mortality

## Abstract

**Background: **COVID-19 disease is accompanied by derangement of coagulation with a risk of fatal thromboembolic formation. COVID-19 patients are among those indicative for heparin treatment. Increased heparin administration among COVID-19 patients increased heparin induced-thrombocytopenia's risk with/without thrombocytopenia.

**Case presentation: **We present a 71-year-old male patient who came to the emergency room (ER) with a COVID-19 clinical manifestation followed by positive PCR nasopharyngeal swab result. He was assessed to have acute respiratory distress syndrome (ARDS), as shown by rapid progression of hypoxemic respiratory failure and bilateral pulmonary infiltrate. He was then treated with moxifloxacin, remdesivir, dexamethasone, unfractionated heparin (UFH) pump, and multivitamins. During admission, his respiratory symptoms got worse, so he transferred to the ICU for NIV support. On the ninth day of admission, he had gross hematuria followed by a rapid fall of platelet count. We used two different scoring systems (4Ts and HEP scoring system) to confirm the diagnosis of heparin-induced thrombocytopenia (HIT). Following the discontinuation of UFH injection, the thrombocyte continued to rise, and hematuria disappeared.

**Conclusion: **Heparin-induced thrombocytopenia is associated with an increased risk of severe disease and mortality among COVID-19 patients. The differential diagnosis of HIT could be difficult as thrombocytopenia can also be caused by the progression of infection. We use two scoring systems (4Ts and HEP scoring) in order to help us managing the patient. These could improve the outcomes, thus avoiding morbidity and mortality.

## Background

The outbreak of the novel-coronavirus SARS-CoV-2 was first identified in Wuhan, China, in January 2020. This was later classified as a pandemic because it affected more than 114 countries with more than two million cases. The infection manifest as fever, dry cough, myalgia, diarrhea, and anosmia. Acute respiratory distress syndrome (ARDS) canbe a manifestation of severe cases, which is shown by rapid progression of hypoxemic respiratory failure and bilateral pulmonary infiltrate. Hypercoagulability often complicates COVID-19 infection as part of its patophysiology and some may progress into venous and arterial thrombosis. ^1^ The laboratory results of COVID-19 patients show an elevated D-dimer concentration, increased fibrin degradation products (FDP), and lower antithrombin levels. These are the reasons for anticoagulation administration among COVID-19 patients. Heparin is the agent of choice for anticoagulation, especially in those with severe COVID-19 manifestation. ^2– 5^

Although heparin is commonly used in COVID-19 patients, its side effects cannot be forgotten. The most avoided side effect, which is potentially fatal with 20% mortality rates, is heparin induced thrombocytopenia (HIT) with/without thrombocytosis. This is caused by the formation of antibodies (IgG) against the complex of platelet factor 4 (PF4) and heparin. The PF4/IgG antibodies complex can activate platelets, which can cause catastrophic thrombosis. It can occur within 5–10 days of heparin therapy in 0.5–3% of patients. It can also develop rapidly within 24 hours after re-exposure of heparin in some patients with a recent heparin administration history. ^2, 6, 7^ An increasing incidence of HIT occurs among COVID-19 patients, explained by exacerbated immune reactions and probably by an increased release of PF4, linked to platelet activation. However, the underlying pathophysiology of increased HIT risk and thrombosis risk in COVID-19 patients is not yet well understood. ^8^ We present a HIT case with severe thrombocytopenia followed by gross hematuria manifestation in a COVID-19 patient.

## Case presentation

A 71-year-old male patient presented to the emergency room (ER) with a chief complaint of dry cough for one week followed by dyspnea and febrile that had increased three days before admission. He had a fever one week prior admission that relieved with sacetaminophen, and no other complaints were reported. He had no contact history with confirmed COVID-19 patients. He has history of diabetes mellitus, hypertension, and myocardial infarction. He previously consumed aspirin, bisoprolol, lisinopril, diltiazem, atorvastatin and subcutaneous insulin.

His blood pressure (BP) was 132/75 mmHg, heart rate (HR) 79 beats per minutes (bpm) regular, and respiratory rate (RR) of 28 breaths per minute, with oxygen saturation of 85% (free air) that improved to 95% with a non-rebreathing oxygen mask (NRM) 15 Liters per minute. His physical examination showed no increased jugular venous pressure, bilateral lung crackles, pleural friction rub, and leg edema.

Comprehensive evaluations were performed, including ECG, COVID-19 antigen swab, laboratory examination, and chest radiography. His initial ECG showed normal sinus rhythm of 79 bpm, left axis deviation, with inferior OMI. Laboratory data showed increased CRP (195.3 mg/L), hypokalemia (3.2 mEq/L), respiratory failure, and respiratory acidosis. Chest radiography showed cardiomegaly and bilateral pneumonia. COVID-19 antigen swab was positive. Thus patient and family were consent for isolation and tracing procedure. As his blood gas showed respiratory failure with severe ARDS ( *P*aO _2_/ *F*iO _2_ 82 mmHg), we planned on invasive ventilation, however the procedure was postponed due to unavailability of ventilator machine and intensive care unit overload.

He was then started on antibiotic treatment with moxifloxacin, antiviral therapy with remdesivir, dexamethasone, continuous infusion of unfractionated heparin (UFH) multivitamins and his daily medication (insulin, aspirin, lisinopril, beta-blockers, nitrates, and atorvastatin). Tocilizumab 400 mg was also given on the second day of admission and continued for 5 days. His vital signs and laboratory examinations were routinely monitored.

During the sixth day of admission, his oxygen saturation deteriorated. He was then put on non-invasive ventilation (NIV) with slightly better oxygen saturation (92-94%). His symptoms showed gradual improvement in several days. His laboratory examination on the tenth day showed leukocytosis, thrombocytopenia, increased neutrophil–lymphocyte ratio (NLR), increased D-Dimer. No prolonged hemostatic values were found. Plasma prothrombin time (PPT) was 20 sec, and activated partial thromboplastin time (APTT) was 38.6 sec.

**Figure 1.**
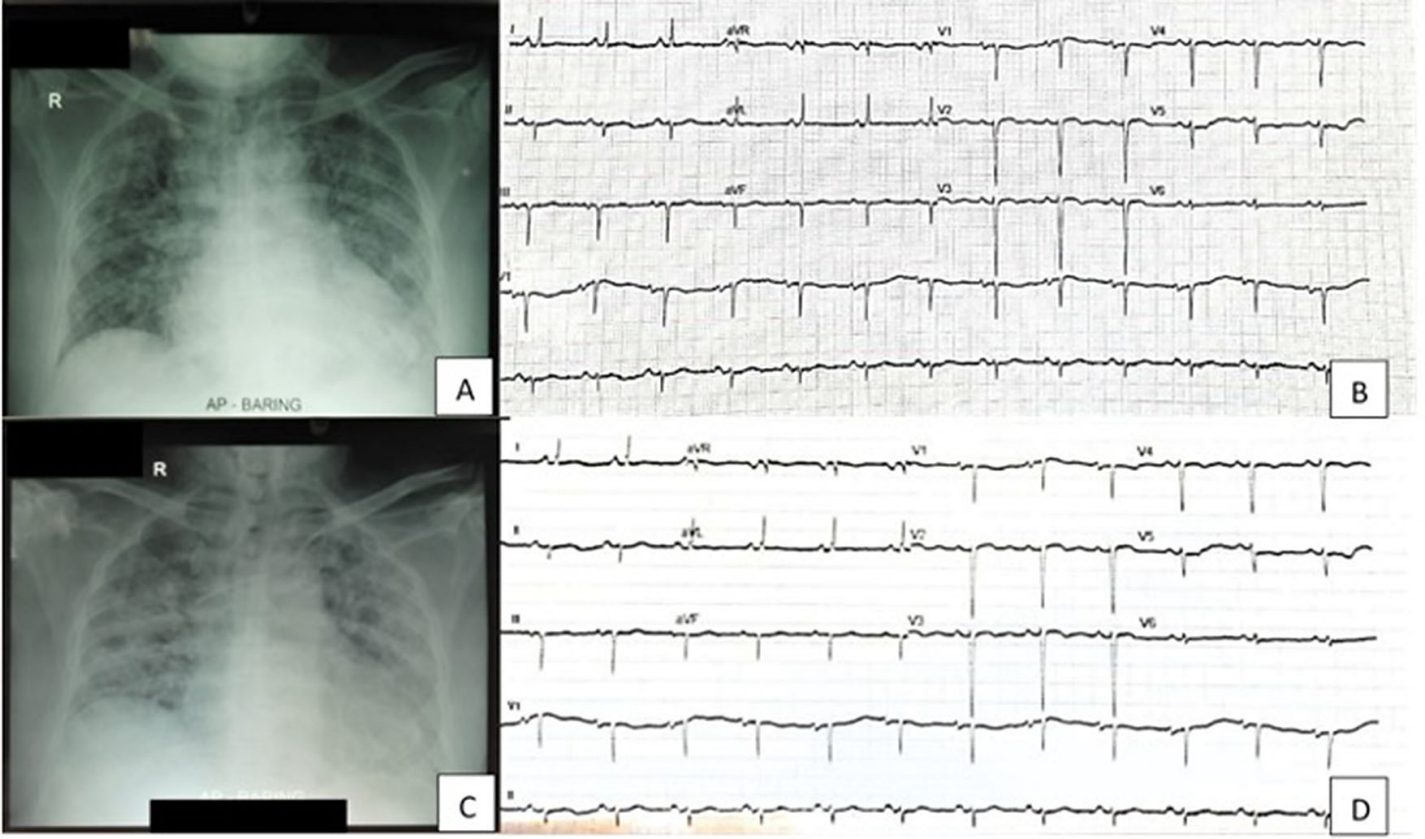
Radiology and electrocardiography (ECG) comparison of patient on first day admission (A, B) and ninth day admission (C, D).

On the ninth day of admission, the patient reported gross hematuria, at that time he was still on unfractionated heparin therapy within targeted APTT. Laboratory data showed significantly reduced thrombocytes (40,000 mg/L). Urine examination showed gross hematuria with leukocyturia (10–15 cells/field), nitrituria, and proteinuria (2+). The patient underwent ECG and chest radiography evaluation, which showed no significant changes ( Figure 1).

**Figure 2.**
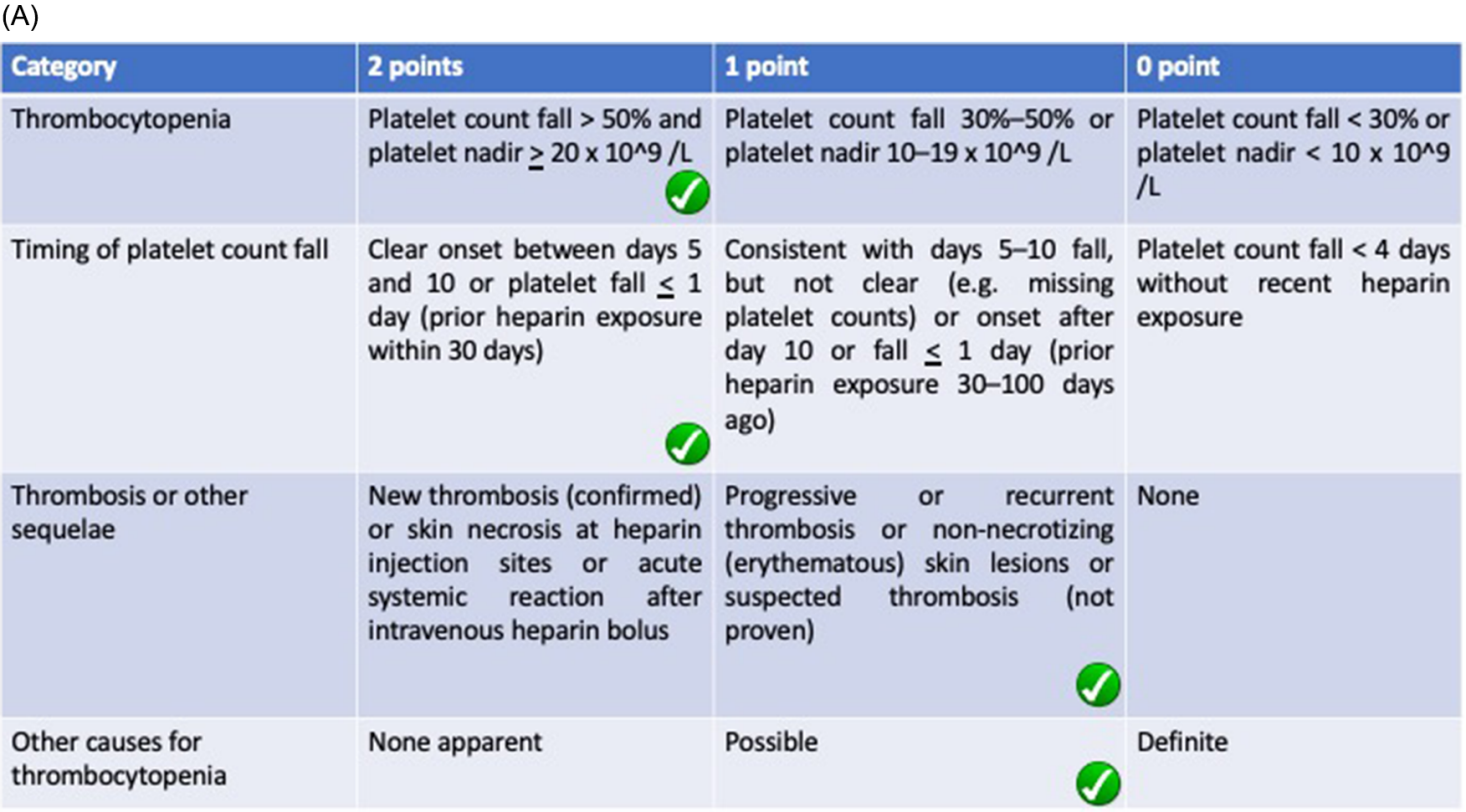

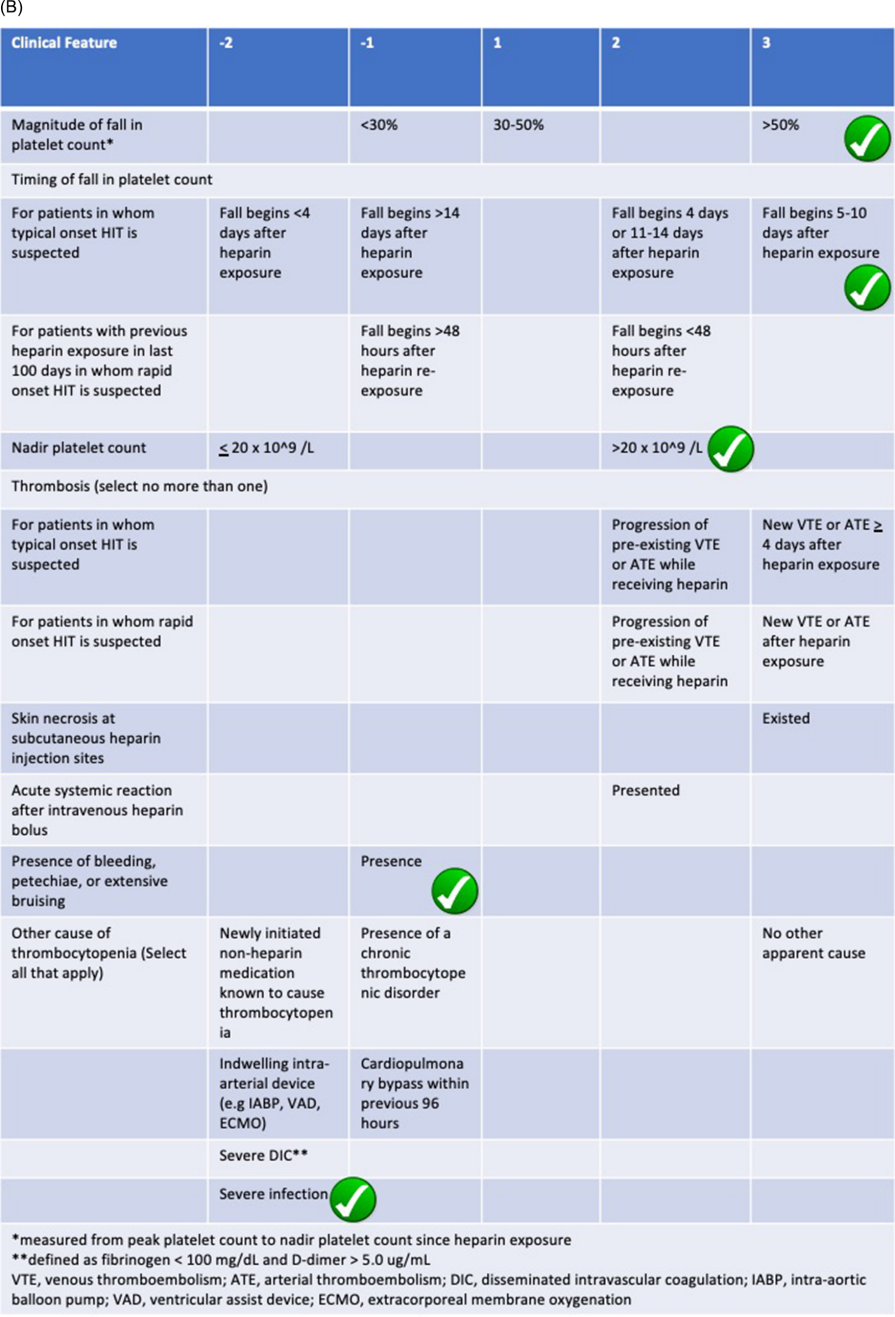
Checklist of scoring system used in measuring HIT probability using (A) 4Ts scoring system showing 6 point and (B) HEP scoring system showing 5 point. Both measurements show high probability of HIT in this patient.

Heparin was thought to be the underlying cause of thrombocytopenia in this patient. Since heparin antibody was not routinely checked in the developing country, we choose HIT’s scoring system as an alternative. The scoring system shows 4Ts of 6 and HEP Scoring system of 5 ( Figure 2A-B). Both 4Ts and HEP scoring indicate a high-risk probability for HIT, hence the anticoagulant was switched to rivaroxaban 20 mg twice daily. The symptoms were then improved with the absence of hematuria after 2 days. The thrombocyte evaluation showed normalization to 177,000 on the eleventh day. The patient was transferred back to the low care and discharged after 13 days.

## Discussion

We report a patient presented to the ED with clinical manifestation of COVID-19 with severe ARDS ( *P*aO _2_/ *F*iO _2_ of 82 mmHg). COVID-19 patient is among those indicative for heparin treatment. Low-molecular-weight heparin is used as prophylaxis for the thromboembolic complication of COVID-19, while unfractionated heparin is commonly used in severe manifestations.

A routine evaluation of both physical and laboratory examination is needed to evaluate treatment and side effects of infection and therapy of COVID-19. A fatal side effect that can occur during heparin treatment is HIT. HIT is characterized by a fall of platelet count with/without a sign of bleeding, arterial, or venous thrombosis during heparin administration and disappears equally quickly once the heparin is withdrawn. The widely known diagnostic criteria for HIT includes (1) HIT antibodies to PF4-heparinoid complexes, (2) HIT antibodies-mediated platelet activation, (3) progressive platelet fall 40-50% from baseline, (4) thrombocytopenia occurs within 5–10 days after initiation or 24–48 hours after re-exposure of heparin, and (5) thrombosis occurs in patients treated with heparin. It should also be suspected of an unexplained fall in platelet count by 50%, skin lesion at the heparin injection site, and systemic reaction to heparin injection. Thrombocytopenia in HIT is usually moderate to severe, with the fall of platelet rarely <100,000 platelets/μL. It has been reported that 30–60% of patients can suffer venous events while 15–20% can suffer arterial events while bleeding manifestation is rare. ^2, 5, 9, 10^

The differential diagnosis of HIT could be difficult among COVID-19 patients as thrombocytopenia can also be caused by infection progression. Disseminated intravascular coagulopathy (DIC) can complicate the septic status of COVID-19, called sepsis-induced coagulopathy. Thrombocytopenia is associated with an increased risk of severe disease and mortality among COVID-19 patients. On the other hand, heparin exposure is correlated with severe thrombocytopenia, suggesting that HIT may be responsible for severe thrombocytopenia in COVID-19. PF4/H antibodies or a scoring system makes the presumptive diagnosis of HIT. The measurement of PF4/H is not commonly checked in our center, so we use the scoring system to measure the possibility of HIT. ^2, 5, 7, 9, 11^

Our patient experienced a rapid fall of thrombocytes on a ninth day following gross hematuria manifestation. The clinical suspicion laid on HIT as it is known to be a fatal side effect of heparin administration. We use two scoring systems, which showed a high probability of HIT in our patient using the 4Ts (5 points) and HEP scoring (5 points) system ( Figure 2). The use of two different methods reduces the possibility of overdiagnosis since improper anticoagulation in COVID-19 patients can lead to worse prognosis. The 4Ts scoring system valued thrombocytopenia, timing of platelet fall, thrombosis (or other sequelae of HIT), and the absence of other thrombocytopenia cause (medication, DIC) in which the fourth indicator is subjective to the discretion of the observer. On the other hand, the HEP scoring system listed infection as a marker of thrombocytopenia which is found in COVID-19 patients. The clinician can confidently reduce the use of alternative anticoagulation due to false diagnosis in clinically oriented HIT assessment using the HEP scoring system.

**Figure 3.**
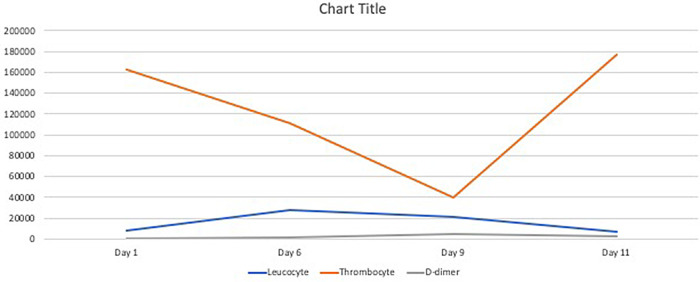
Summary of laboratory results.

When isolated HIT is suspected clinically, the discontinuation of heparin-based therapy is recommended. Clinicians shall consider the initiation of other anticoagulant agents using direct thrombin or factor Xa inhibitors to prevent thrombus formation. ^7, 9^ Rivaroxaban was chosen as substitute agent as it was structurally different from heparin and no cross reactivity was found to heparin-PF4 antibodies. Following the discontinuation of heparin injection and switching to rivaroxaban, the thrombocyte continued to rise, and the hematuria resolved ( Figure 3).

## Conclusion

There has been an escalation of heparin usage during the outbreak of SARS-CoV-2 infection as it is a known risk of fatal thromboembolic formation. HIT is a rare but fatal complication following heparin administration. As the progression of COVID-19 also shows thrombocytopenia, it is somehow ambiguous to perform discontinuation of heparin. Moreover, the measurement of PF4/H antibodies is not available in most of the centers. It makes the clinical assessment of HIT critical to risk-stratify the need to stop heparin as it also led to poor prognosis. The use of 4Ts scoring system is subjective to the observer's discretion to determine the involvement of other thrombocytopenia. However, the HEP scoring system has multiple values that help physicians determine the HIT risk probability. We used two different scoring systems (4Ts and HEP scoring systems) that show a high HIT probability. As important as diagnosing HIT, heparin product must be discontinued and substitute anticoagulant with no heparin profile is recommended based on indication.

## Declaration

This case report does not require ethical approval as it is not human or animal research.

## Consent

Written informed consent for publication of their clinical details and/or clinical images was obtained from the patient.

## Data Availability

All data underlying the results are presented within the manuscript, and no additional source data are required.
