# Supplementary material for: Case Report: Heparin-induced thrombocytopenia during COVID-19 outbreak: the importance of scoring system in differentiating with sepsis-induced coagulopathy
Source: F1000Res. 2021 Aug 26;10:469. Originally published 2021 Jun 14. [Version 2] doi: 10.12688/f1000research.52425.2 (PMC8356265; doi:10.12688/f1000research.52425.2)
Supplement: Peer review history

# Peer review: Reviewer response for version 1

**DOI:** 10.5256/f1000research.55699.r90612

**Published:** 2021-08-09

**Authors:** Anggoro Budi Hartopo

**Affiliations:** 1 Department of Cardiology and Vascular Medicine, Faculty of Medicine, Public Health and Nursing, Dr. Sardjito Hospital, Universitas Gadjah Mada, Jogjakarta, Indonesia

The authors report a case of COVID-19 pneumonia with HIT complication and finally can survive the disease. The emphasis of recognition of HIT, by two scoring system assessment, and proper discontinuation of heparin (UFH?) benefit the patient survival. 

 However several concerns were addressed regarding the case presentation, as follows:

1. "Tocilizumab 400 mg was also given on the second day before admission", is it true that **before hospital admission** the tocilizumab already be given?
2. What kind heparin is used in this case? Continuous infusion of unfractionated heparin or subcutaneous LMWH? Please describe clearly the heparin type. 
3. "Since heparin antibody was not routinely checked in the developed country," what developed country this refers to? 
4. The use of 4Ts and HEP scoring must be reported in the Case presentation which recite the patient situation and management given, not in the Discussion session. In the Case presentation, there is no statement/narration about the use of these scores during patient management. 
5. Author switch to rivaroxaban as alternative anticoagulant in HIT replacing heparin, please give comment and reference/guideline which support this management.

Are enough details provided of any physical examination and diagnostic tests, treatment given and outcomes?

Yes

Is the case presented with sufficient detail to be useful for other practitioners?

Partly

Is sufficient discussion included of the importance of the findings and their relevance to future understanding of disease processes, diagnosis or treatment?

Yes

Is the background of the case’s history and progression described in sufficient detail?

Partly

Reviewer Expertise:

Cardiovascular medicine

I confirm that I have read this submission and believe that I have an appropriate level of expertise to confirm that it is of an acceptable scientific standard.

# Response

**Published:** 2021-08-10

**Authors:** Louisa Fadjri Kusuma Wardhani

**Affiliations:** Airlangga University, Indonesia

The authors report a case of COVID-19 pneumonia with HIT complication and finally can survive the disease. The emphasis of recognition of HIT, by two scoring system assessment, and proper discontinuation of heparin (UFH?) benefit the patient survival. 

 However several concerns were addressed regarding the case presentation, as follows:

- "Tocilizumab 400 mg was also given on the second day before admission", is it true that **before hospital admission** the tocilizumab already be given? 
  - Thank you for your correction. Tocilizumab was given in the second day admission. The following correction has been submitted in the case presentation section.
- What kind heparin is used in this case? Continuous infusion of unfractionated heparin or subcutaneous LMWH? Please describe clearly the heparin type. 
  - Thank you for your correction. We used unfractionated heparin (UFH) pump at first. We have been decribed it in the revision format.
- "Since heparin antibody was not routinely checked in the developed country," what developed country this refers to?
  - The correct statement is “Since heparin antibody was not routinely checked in the developing country”  since its not applicable in our country (Indonesia). 
- The use of 4Ts and HEP scoring must be reported in the Case presentation which recite the patient situation and management given, not in the Discussion session. In the Case presentation, there is no statement/narration about the use of these scores during patient management.
  - Thank you for your kind suggestion. We have added the use of both scoring system in our case presentation section. 
- Author switch to rivaroxaban as alternative anticoagulant in HIT replacing heparin, please give comment and reference/guideline which support this management.
  - The drug of choices for whom suspected for HIT is argatroban and danaparoid. There is several recommendations regarding secondary prevention of HIT using rivaroxaban and fondaparinux (A Cuker, 2010; Lori Ann Linkins, BMJ 2015). Since the availability of some drug is limited and patient with ongoing hematuria, We prefer rivaroxaban as an exchange. Moreover, the use of rivaroxaban twice daily has non-inferior efficacy than those with fondaparinux (ESC Guidelines for Pulmonary Embolism).

# Peer review: Reviewer response for version 1

**DOI:** 10.5256/f1000research.55699.r87581

**Published:** 2021-06-30

**Authors:** Alan Fong

**Affiliations:** 1 Department of Cardiology, Sarawak Heart Centre, Kota Samarahan, Malaysia

This case report illustrates a clinical scenario that has to be identified early. Early risk stratification of the unwell patient and institution of an aggressive management plan will improved outcomes. This has been discussed in this draft, which is commendable. Therefore, this case report is relevant during this COVID pandemic.

 However, language editing is recommended, for better presentation of this manuscript in your journal.

 Therefore, minor revision, and then suitable for indexing.

Are enough details provided of any physical examination and diagnostic tests, treatment given and outcomes?

Yes

Is the case presented with sufficient detail to be useful for other practitioners?

Yes

Is sufficient discussion included of the importance of the findings and their relevance to future understanding of disease processes, diagnosis or treatment?

Yes

Is the background of the case’s history and progression described in sufficient detail?

Yes

Reviewer Expertise:

Cardiovascular diagnostics

I confirm that I have read this submission and believe that I have an appropriate level of expertise to confirm that it is of an acceptable scientific standard.

# Response

**Published:** 2021-08-10

**Authors:** Louisa Fadjri Kusuma Wardhani

**Affiliations:** Airlangga University, Indonesia

This case report illustrates a clinical scenario that has to be identified early. Early risk stratification of the unwell patient and institution of an aggressive management plan will improved outcomes. This has been discussed in this draft, which is commendable. Therefore, this case report is relevant during this COVID pandemic.

 However, language editing is recommended, for better presentation of this manuscript in your journal.

 Therefore, minor revision, and then suitable for indexing.

Thank you for your kind suggestion and comment. We have reviewed our manuscript and restructured with a better language editing
